# Effects of spray-dried plasma on nutrient digestibility, fecal metabolites, microbiota, and immune and inflammatory biomarkers in adult dogs

**DOI:** 10.1093/jas/skaf373

**Published:** 2025-11-03

**Authors:** Julio C Mioto, Patricia M Oba, Joy M Campbell, Maria R C de Godoy

**Affiliations:** Department of Animal Sciences, University of Illinois, Urbana, IL 61801; Department of Animal Sciences, University of Illinois, Urbana, IL 61801; APC LLC., Ankeny, IA 50021; Department of Animal Sciences, University of Illinois, Urbana, IL 61801; Division of Nutritional Sciences, University of Illinois, Urbana, IL 61801

**Keywords:** canine nutrition, digestibility, immunity, metabolism, microbiota, spray-dried plasma

## Abstract

Spray-dried plasma (SDP) is a high-quality protein source with functional properties that support gut health, immune function, and digestibility. Although its benefits have been demonstrated in various animal species (e.g., swine, poultry, rodents, and fish), further research is needed to understand its specific effects in extruded diets and canine health. This study evaluated the effects of incorporating SDP from swine, in partial replacement of chicken meal, into extruded diets on nutrient digestibility, fecal metabolites, microbiota composition, serum chemistry, and inflammatory and immune biomarkers in adult dogs. Extrusion processing parameters were also evaluated in diets containing increasing levels of SDP. Four dietary treatments were formulated: a Control diet without SDP, a diet with 4% SDP applied as a coating (4% coating diet), and diets with 4% or 8% SDP included within the kibble matrix prior to extrusion. Twelve adult Beagles (*n* = 12) were assigned to the four dietary treatments in a replicated 4 × 4 Latin Square design, with each diet fed for 21 d following a 7-d adaptation period. All diets were well accepted by the dogs. Feeding 8% SDP maintained overall nutrient digestibility but reduced total dietary fiber digestibility (*P* < 0.05). Fecal concentrations of short-chain fatty acids, particularly propionate, were higher in dogs fed the 8% SDP diet (193.8 µmol/g) compared with the Control diet (126.8 µmol/g; *P* < 0.05). Additionally, a linear reduction in protein fermentation byproducts, mainly indole metabolites, was observed, suggesting a beneficial shift in microbial metabolism (*P* < 0.05). Inclusion of SDP also modulated fecal microbiota composition, with changes across several genera and increases in beneficial genera such as *Lactobacillus*, *Streptococcus*, and *Catenibacterium*, while also affecting beta-diversity (*P* < 0.05). Serum chemistry analysis showed lower blood urea nitrogen in dogs fed the 4% Coating SDP (17.8 mg/dL), 4% SDP (18.0 mg/dL), and 8% SDP (16.3 mg/dL) diets compared with the Control diet (19.8 mg/dL; *P* < 0.05), suggesting improved protein metabolism. Fecal IgA concentrations were higher in dogs fed 8% SDP than the Control diet, indicating potential immunomodulatory effects that may contribute to improved intestinal homeostasis (*P* < 0.05). These findings support the potential of SDP as a functional ingredient in canine diets, contributing to gut health, metabolic regulation, and immune function in extruded pet food.

## Teaser

Incorporating spray-dried plasma into extruded dog diets influenced nutrient digestibility and modulated gut microbiota, suggesting potential health benefits for adult dogs.

## Introduction

The growing interest in functional ingredients for pet foods reflects a broader trend toward improving animal health and well-being through diet. This trend is driven by the strong emotional bond between humans and their companion animals, leading pet owners to seek ways to enhance their pets’ health, quality of life, and longevity (de Godoy et al., 2013). The development of functional pet food products has focused on improving nutritional adequacy and supporting specific physiological and health benefits, such as digestive health and immune modulation. The growing demand for high-quality raw materials, along with challenges posed by the supply chain and the need for more sustainable ingredients, has influenced the pet food industry (Beaton, 2021; Kemin, 2024), where there is increasing interest in protein sources that not only fulfill nutritional requirements but also provide functional benefits, supporting health while minimizing environmental impact (Vasconcellos et al., 2023).

Among these, spray-dried plasma (SDP) has emerged as a promising ingredient to address these industry demands. SDP is a dry functional ingredient obtained from the blood of healthy animals (e.g., bovine, poultry, and swine) collected in slaughterhouses and processed into a spray dryer to preserve the functional characteristics of the proteins (Howell and Lawrie, 1983; Parés et al., 1998; Campbell et al., 2019; Blázquez et al., 2020). Nutritionally, SDP contains 70%–80% crude protein, around 1% fat, 5%–9% ash, and provides approximately 5 kcal/g of gross energy. It is particularly rich in essential amino acids such as lysine, leucine, threonine, valine, and phenylalanine + tyrosine, whereas isoleucine and the sulfur amino acids (methionine + cystine) are limiting compared with whole egg powder, considered a premium protein source in the pet food industry (Kazimierska and Biel, 2023). It is a highly nutritious ingredient, rich in essential amino acids and bioactive compounds, including immunoglobulins, albumin, fibrinogen, growth factors, biologically active peptides, transferrin, and enzymes (Bah et al., 2013; Pérez-Bosque et al., 2016b; Miró et al., 2017; Campbell et al., 2019; Balan et al., 2021). These components contribute to its functional properties, such as prebiotic-like (Miró et al., 2017; Moretó et al., 2020) and anti-inflammatory effects (Pérez-Bosque et al., 2016a; Moretó et al., 2020), exerting systemic and intestinal benefits independent of their nutritional value (Campbell et al., 2019).

Spray-dried plasma is widely used in livestock diets due to its functional properties, which enhance nutrient digestibility, support intestinal integrity, modulate immune responses, and improve growth performance in farm animals (Torrallardona, 2009; Boyer et al., 2015; Kim et al., 2022). These benefits have been observed in various species, including fish (Fernández-Alacid et al., 2022; Xu et al., 2022), poultry (Belote et al., 2021; Granghelli et al., 2023), and pigs (Zhe et al., 2021; Kim et al., 2022; Bailey et al., 2025).

Although the benefits of SDP have been extensively studied in livestock and aquaculture, its use in companion animals, particularly dogs, has been less explored. However, some studies suggest that SDP may exert beneficial effects on canine nutrition. Low inclusion of SDP (2%) in dry dog food kibble, either before or after extrusion, has been shown to improve dry matter, crude fiber, and total dietary fiber digestibility while reducing fecal dry matter excretion (Quigley et al., 2004). Additionally, dogs fed a diet containing 12% SDP showed an increased number of total leukocytes and higher concentrations of total blood proteins and albumin (de Andrade et al., 2019). Moreover, SDP has been associated with immunomodulatory effects in dogs, contributing to gut health and immune function. Rodriguez et al. (2007) showed that the inclusion of SDP in diets can influence immune parameters, such as increasing serum and mucosal immunoglobulin levels (IgA and IgG) in dogs and cats, suggesting that it supports mucosal immunity by modulating immune responses.

Despite these findings, a key consideration when incorporating SDP in pet food is that the high temperatures and pressure applied during the extrusion and sterilization processes can potentially denature proteins present in SDP (Rodríguez et al., 2016). Nevertheless, the improved digestibility observed when SDP is added before extrusion suggests that its benefits may not be solely dependent on intact functional proteins (Rodríguez et al., 2016), which indicates that certain bioactive components in SDP may withstand processing, preserving their potential effects on nutrient utilization and gut health.

Therefore, the purpose of this study was to determine the effects of SDP inclusion in extruded diets for adult dogs by evaluating the impact of incorporating SDP at 4% as a kibble coating and at 4% or 8% within the kibble matrix before extrusion, assessing its effects on nutrient digestibility, fecal metabolites, microbiota composition, serum health markers, and inflammatory and immune biomarkers. We hypothesize that the inclusion of SDP in extruded diets at different levels and incorporation methods (coating vs. pre-extrusion) will influence nutrient digestibility, with coating expected to better preserve bioactive. Additionally, we expect that SDP diets will modulate fecal microbiota composition by increasing short-chain fatty acids and reducing proteolytic metabolites and will affect immune parameters, specifically increasing fecal IgA and reducing inflammatory biomarkers (i.e., calprotectin), thereby supporting mucosal immunity, intestinal homeostasis, and overall health in adult dogs.

## Materials and Methods

All animal procedures were approved by Kennelwood Inc., Champaign, IL. Animal Care and Use Committee prior to animal experimentation. All methods were performed in accordance with the United States Public Health Service Policy on Humane Care and Use of Laboratory Animals.

### Experimental diets

Four treatment diets were formulated to have similar ingredient compositions except for the main protein source (Table 1). The Control diet was formulated with chicken meal low ash (CM) as the primary protein source and brewers rice as the primary starch source. SDP from swine, provided by APC LLC, Ankeny, IA, USA, was used to partially substitute CM in manufacturing extruded diets for adult dogs. The diets were as follows, 1) Control: 0% SDP supplementation; 2) 4% SDP Coating: 4% of substitution SDP of CM diet added only at the coating step; 3) 4% SDP: 4% of substitution SDP of CM diet prior to extrusion; and 4) 8% SDP: 8% substitution of SDP of CM diet prior to extrusion. All diets were formulated to meet or exceed the AAFCO (2024) recommendation for adult dog maintenance and were extruded at Wenger Pilot Plant at Sabetha, KS, USA. Prior to extrusion, all diets were ground using a 1.19 mm screen, resulting in an average particle size of approximately 350–370 µm. Processing conditions were maintained as stable as possible to produce viable extruded diets to be used in animal feeding trials. Extrusion processing conditions of experimental diets are shown on Table 2. Following extrusion and drying, all kibbles were coated in a horizontal paddle mixer. All diets received the same fat plus liquid palatant application to standardize coating process and lipid levels across treatments. In the 4% SDP Coating diet, the SDP powder replaced an equivalent portion of CM and was applied together with the fat plus liquid palatant during coating.

**Table 1.** Ingredient composition of control diet and diets containing 4% or 8% of spray-dried plasma (SDP) for adult dogs.

Item, %	Diets			
	Control	4% SDP Coating	4% SDP	8% SDP
**Brewers rice**	39.63	40.05	40.05	40.08
**Chicken meal**	33.20	27.65	27.65	22.15
**Chicken fat**	7.76	8.39	8.39	9.02
**Corn**	8.00	8.00	8.00	8.00
**Spray-dried whole egg**	4.00	4.00	4.00	4.00
**Spray dried plasma**	0.00	4.00	4.00	8.00
**Powdered cellulose**	3.00	3.00	3.00	3.00
**Sugar beet pulp**	2.00	2.00	2.00	2.00
**Palatant**	1.00	1.00	1.00	1.00
**Potassium chloride**	0.55	0.55	0.55	0.59
**Limestone**	0.00	0.50	0.50	1.30
**Sodium chloride**	0.30	0.30	0.30	0.30
**Choline chloride**	0.20	0.20	0.20	0.20
**Mineral premix^1^**	0.18	0.18	0.18	0.18
**Vitamin premix^2^**	0.18	0.18	0.18	0.18

1 Provided per kilogram diet: 18.0 mg Cu (as CuCO_3_), 1.8 mg I (as KIO_3_), 135.0 mg Fe (as C_6_H_5_FeO_7_), 18.0 mg Mn (as MnCO_3_), 0.396 mg Se (as Na_2_SeO_3_), 180.0 mg Zn (as ZnCO_3_), 0.0038 mg Co (as CoSO_4_).

2 Provided per kilogram diet: 18,000 IU vitamin A acetate, 2,700 IU vitamin D_3_, 144 IU vitamin E acetate, 2.16 mg menadione sodium bisulfite, 0.108 mg biotin, 0.115 mg cyanocobalamin, 1.08 mg folic acid, 124.2 mg nicotinic acid, 50.4 mg calcium pantothenate, 30.6 mg pyridoxine-HCl, 30.6 mg riboflavin, 30.6 mg thiamin HCl.

**Table 2.** Average twin screw extruder processing conditions for experimental diets containing 4% or 8% of spray-dried plasma (SDP).

		Diets			
Measurement	Unit	Control	4% SDP Coating	4% SDP	8% SDP
**Raw material**					
** Dry recipe density**	kg/m^3^	452	452	452	452
** Dry recipe rate**	kg/h	496	497	495	497
** Feed screw speed**	rpm	68	65	62	62
**Preconditioner**					
** Steam flow**	kg/h	29	29	30	30
** Water flow**	kg/h	100	100	101	99
**Extruder**					
** Speed**	rpm	475	515	515	515
** Motor load**	%	45	47	48	44
** Knife speed**	rpm	1,925	2,000	2,000	2,000
** Steam flow**	kg/h	0	0	0	0
** Water flow**	kg/h	19	19	19	19
** Zone 1 temperature (Preconditioner Discharge)**	°C	61	59	60	59
** Zone 2 temperature**	°C	70	68	70	69
** Zone 3 temperature**	°C	80	79	79	79
** Zone 4 temperature**	°C	84	85	85	84
** Zone 5 temperature**	°C	99	101	102	100
**Dryer**					
** Retention time-pass 1**	min	15	15	15	15
** Retention time-pass 3**	min	7.07	7.07	7.07	7.07
** Retention time-total**	min	22.07	22.07	22.07	22.07
** Dryer temperature-zone 1**	°C	109	110	109	109
** Dryer temperature-zone 2**	°C	78	79	77	76
** Dryer temperature-zone 3**	°C	105	101	94	94
**Final product**					
** Extruder discharge density**	kg/m^3^	420	420	420	424
** Kibble diameter**	mm	12	11.7	12.1	11.9
** Kibble length**	mm	4	3.6	4	4

### Chemical analysis

All treatment diets and dried fecal samples were ground with a Wiley mini mill (Thomas Scientific, Swedesboro, NJ) through a 1 mm screen. All experimental diets, ingredients, and dried feces were analyzed for dry matter (DM), ash, organic matter (OM), acid hydrolyzed fat (AHF), crude protein (CP), total dietary fiber (TDF), and gross energy (GE). In addition, diets were also analyzed for soluble and insoluble fiber content. Dry matter and ash content of the diets and ingredients were determined in duplicates according to AOAC (2006; methods 934.01 and 942.05). Total nitrogen values were determined according to AOAC (2006; method 992.15) with CP calculated from Leco (TruMac N, Leco Corporation, St Joseph, MI). Concentration of AHF was determined according to AOAC (2006; method 954.02) with using 3N HCl hydrolysis (Ankom HCI Hydrolysis System; Ankom Technology) followed by crude fat extraction using petroleum ether (Ankom XT15; Ankom Technology). Gross energy was determined through bomb calorimetry (Model 6200, Parr Instruments Co., Moline, IL). Total dietary fiber was analyzed according to Prosky et al. (1992).

### Canine study and experimental design

Twelve adult beagles (6 females, mean age 3.0 ± 0.50 yr, mean body weight 9.5 ± 0.52 kg, mean body condition score 5 ± 0.28, and 6 males, mean age 3.5 ± 0.56 yr, mean body weight 9.5 ± 0.80 kg, mean body condition score 5 ± 0.37) were included in the study with a replicated 4 × 4 Latin Square design. In the beginning, the dogs had a 7-d adaptation period, in which all the dogs consumed the Control diet. After the adaptation period, dogs were stratified to 1 of the 4 treatment diets and balanced by sex. Dogs were fed their assigned diets for 21 d per experimental period (4 total). Fresh fecal collection was conducted at the end of the adaptation period (day 0/baseline) and on days 14 and 21 of each period for evaluating fecal metabolites, microbiota, and inflammatory and immune biomarkers. The total fecal collection was conducted during the last 4 d of the treatment period, and a fresh fecal sample was also collected during this time. Fasted blood samples were collected on days 0 and 21 of each period to evaluate health status. During collections, 2 mL of blood was placed in EDTA vacutainer tubes, and 8.5 mL was placed in serum separator tubes (Becton, Dickinson and Company, Franklin Lakes, NJ). Blood samples were sent to the Clinical Pathology Laboratory at the College of Veterinary Medicine at the University of Illinois (Urbana, IL) for serum chemistry and complete blood count analyses.

The dogs were housed individually in wired kennels at Kennelwood (Champaign, IL), allowing nose to nose interaction with adjacent dogs and visual contact with all dogs in a temperature-controlled room. Each kennel provided an indoor area measuring 0.9 m × 1.44 m (1.30 m^2^), with access to an outdoor run measuring 0.9 m × 2.4 m (2.16 m^2^). Feeding occurred once a day at 1,300–1,500. Diets were weighed and recorded for each feeding. Dogs always had free access to water and were fed to maintain body weight. Both body weight and body condition score were monitored weekly.

### Fecal collection, preparation, and fermentative metabolites analyses

A fresh fecal sample was collected from each dog within 15 min of defecation on days 0, 14, and 21 during each period. Fresh fecal samples were scored (1 = hard, dry pellets; 2 = hard formed, remains firm and soft; 3 = soft, formed and moist stool; 4 = soft, unformed stool; or 5 = watery, liquid that can be poured), weighed, and measured for pH. Aliquots of the fresh fecal sample were collected for short-chain fatty acids (SCFA), branched-chain fatty acids (BCFA), and ammonia analyses by placing 5 g of feces into a 60 ml Nalgene bottle containing 5 ml of 2 N hydrochloric acid. Phenols/indoles were collected in duplicate by weighing 2 g of feces into identical plastic tubes and covered with Parafilm. Fecal samples for microbiota analysis were placed in 2 ml cryovials. Fecal samples allocated for fermentative end-product analysis were stored at −20°C. Fecal samples allocated for microbiota were stored at −80°C until analyses.

Total feces were collected during the last 4 d in each period. Weight and fecal scores were recorded for each fecal sample, and samples were stored at -20°C until analyzed for macronutrient apparent total tract digestibility (ATTD). Fecal samples were composited by dog within period. The composited fecal samples were dried in a 57°C forced-air oven. Once dried, fecal samples were ground through a 1 mm screen using a Wiley mill (model 4, Thomas Scientific, Swedesboro, NJ) and analyzed in duplicate according to methods described above.

Fecal concentrations of SCFA and BCFA were measured using gas chromatography according to Sunvold et al. (1995). Fecal ammonia concentrations were determined using spectrophotometer according to Chaney and Marbach (1962). Fecal phenol and indole concentrations were analyzed through gas chromatography according to Flickinger et al. (2003).

### Fecal microbiota

Total DNA was extracted from fresh fecal samples using a DNeasy PowerSoil ProKit (QIAGEN, Germantown, MD). Invitrogen Qubit 4 Fluorometer (Thermo Fisher Scientific Inc., Waltham, MA) was used to measure DNA concentration. A Fluidigm Access Array (Fluidigm Corporation, South San Francisco, CA) combined with Roche High Fidelity Fast Start Kits (Roche, Indianapolis, IN) were used to amplify the 16S rRNA gene. The quality of the amplicons’ regions and sizes was confirmed by Fragment Analyzer (Advanced Analytics, Ames, IA). The pooled DNA samples were selected by size on a 1% agarose E-gel (Life Technologies, Grand Island, NY) and extracted using a gel extraction kit (QIAGEN, Germantown, MD). The pooled, size-selected, and cleaned products were then run on an Agilent Bioanalyzer to confirm the appropriate profile and mean size. Full-length 16S PacBio (Pacific Biology, Menlo Park, CA) analysis was performed at Roy J. Carver Biotechnology Center at the University of Illinois. The 16S amplicons were generated with the barcoded full-length 16S primers from PacBio and the 2x Roche KAPA HiFi Hot Start Ready Mix (Roche, Willmington, MA). The amplicons were pooled and converted to a library with the SMRTbell express template prep kit 2.0 (Pacific Biology, Menlo Park, CA) on 1 SMRTcell 8M in the Sequel II using the CCS sequencing mode and a 10hs movie time. The upstream analysis workflow was done according to Ras et al. (2021).

The FASTX toolkit (version 0.0.13) was employed to trim forward reads, and the resulting sequence data was processed using QIIME2 (Caporaso et al., 2011). Raw sequence amplicons were imported into QIIME2 and analyzed for quality control through the DADA2 pipeline (QC value ≥ 20; Callahan et al., 2016). Samples underwent rarefaction to 27,650 reads, followed by taxonomic assignment using the Silva database (Silva 138) 99% operational taxonomic units (Bokulich et al., 2018; Robeson et al., 2021). Alpha diversity was assessed by observed taxa, Chao1, Shannon, Simpson, Inverse Simpson, and Fisher indexes. The rarefied samples were utilized for alpha and beta diversity analyses. Both weighted and unweighted unique fraction metric (UniFrac) distances were used to analyze principal coordinates after converting Amplicon Sequence Variants (ASV) abundances proportions (Lozupone and Knight, 2005). The feature table, rooted tree from reconstructed phylogeny and taxonomy classification were imported from QIIME2 into the R v4.1.2 environment. Further data analysis was conducted using Microbiome v1.16.0 and Phyloseq v1.38.0 R packages (McMurdie and Holmes, 2013). Multidimensional scaling ordination was applied to the chosen beta diversity metrics using the plot ordination function from the Phyloseq package in R. Spearman’s rank correlation coefficient (r) was performed using the microbiome package (version 1.24.0) to assess the correlation between genus taxa and fecal metabolites and characteristics.

### Calprotectin and immunoglobulin A (IgA) analysis

Canine fresh feces were homogenized in phosphate-buffered saline and centrifuged to obtain supernatants, which were analyzed to determine calprotectin and immunoglobulin A (IgA) concentrations using commercially available quantitative sandwich ELISA kits (MyBioSource Inc., San Diego, CA; Calprotectin: Cat. No. MBS030023, detection range 6.25–200 ng/mL, sensitivity 1.0 ng/mL; IgA: Cat. No. MBS018650, detection range 31.2–1,000 μg/mL, sensitivity 5.0 μg/mL). Standards and samples were analyzed in duplicate following the manufacturer’s instructions, and absorbance was measured at 450 nm with results calculated from standard curves.

### Statistical analysis

Data was analyzed using SAS (version 9.4, SAS Institute Inc., Cary, NC). The MIXED procedure was used for digestibility, serum chemistry, metabolites, and other continuous outcomes. Fecal scores, treated as ordinal data, were analyzed using the GLIMMIX procedure. Dietary treatment, period, and collection day were specified as fixed effects, and animal was treated as a random effect. Residual normality was evaluated with the UNIVARIATE procedure and the Shapiro–Wilk test; variables that violated normality assumptions were transformed as needed. Tukey adjustment was applied to control experiment-wise type I error, and significance was declared at *P* < 0.05. Pooled standard errors of the mean (SEM) were obtained from the MIXED or GLIMMIX procedures. Linear contrasts were used to test incremental SDP inclusion prior to extrusion relative to the control diet. In addition, microbiota analyses were conducted using rarefied samples. Alpha diversity was calculated across standard indices, whereas beta diversity (weighted and unweighted UniFrac distances) was tested by PERMANOVA (999 permutations). Differential abundance of phyla and genera was evaluated with ANCOM-BC2 (R package ANCOMBC), using dietary treatment as the fixed factor and applying Benjamini-Hochberg false discovery rate correction. Significance was defined as *P* < 0.05.

## Results

### Chemical composition of diets

Chemical composition was similar among all 4 dietary treatments (Table 3). The DM content ranged from 94.0% to 94.5%, OM concentration ranged between 93.5 and 94.1% on dry matter basis (DMB), CP concentration was around 27.8% to 31.2%, AHF content ranged from 14.1% to 15.2%, TDF content was between 13.3 and 16%, and GE was 5.1 kcal/g across all diets. All diets meet the minimum requirements established by NRC (2006) or nutrient profile suggested by AAFCO (2024) for adult dogs.

**Table 3.** Chemical composition of control diet and diets containing 4% or 8% of spray-dried porcine plasma (SDP) for adult dogs

Item %, DMB^1^	Diets			
	Control	4% SDP Coating	4% SDP	8% SDP
**Dry matter, % **	94.5	94.0	94.0	94.3
** **	**%, DMB^1^**			
**Organic matter, % **	93.5	94.1	94.1	93.8
**Ash, %**	6.5	5.9	5.9	6.2
**Crude protein, % **	31.2	28.4	28.5	27.8
**Acid-hydrolyzed fat, %**	14.6	14.1	14.3	15.2
**Total dietary fiber, %**	16.0	13.8	13.3	14.8
** Soluble dietary fiber**	4.2	3.5	3.1	2.3
** Insoluble dietary fiber**	11.9	10.3	10.2	12.5
**Gross energy^2^, kcal/g**	5.1	5.1	5.1	5.1
**Nitrogen-free extract, %**	31.7	37.8	38.0	36.0
**ME^3^, kcal/g**	3.4	3.5	3.5	3.5

1 DM = dry matter basis.

2 Gross energy was measured using bomb calorimetry.

3 ME = Metabolizable energy estimated using modified Atwater factors (3.5 kcal/g for protein and nitrogen-free extract; 8.5 kcal/g for fat).

### Apparent total tract digestibility of diets

There was no difference (*P* > 0.05) in feed intake, fecal output on either as-is or DM basis among treatment groups, and also for fecal scores (Table 4). The ATTD were similar among the dietary groups (*P* > 0.05) for DM (84.5% to 85.9%), OM (87.6% to 88.4%), CP (85.2% to 85.6%), AHF (95.3% to 95.6%), and digestible energy (4.5 kcal). However, for ATTD of TDF, dogs on the control diet had the highest at 60.6% and 4% SDP group was the lowest at 53.4% (*P* < 0.05).

**Table 4.** Food intake, fecal output, fecal scores, apparent total tract macronutrient, and energy digestibility of dogs fed diets containing 4% or 8% of spray-dried porcine plasma (SDP).

Item	Diets				SEM^2^	*P*-value	Linear contrast^4^
	Control	4% SDP coating	4% SDP	8% SDP			
**Food Intake (g), as is**	1072.3	1083.7	1042.3	1076.0	62.99	0.8126	0.9346
**Food Intake (g), DMB^1^**	1033.0	1044.9	1006.0	1039.1	61.08	0.8163	0.8886
**Fecal Output (g), as is**	519.7	497.7	461.5	561.9	50.53	0.2407	0.3967
**Fecal Output (g), DMB^1^**	159.9	147.7	145.2	156.2	11.69	0.5057	0.7486
**Fecal Score**	3.2	3.3	3.2	3.3	0.09	0.1951	0.0615
**Apparent Total Tract Digestibility**							
**Dry matter, % **	84.5	85.9	85.6	84.9	0.69	0.3213	0.6872
**% DMB**							
**Organic matter, % **	87.6	88.4	88.1	87.7	0.58	0.5993	0.9683
**Crude protein, %**	85.2	85.4	85.4	85.6	0.81	0.9824	0.7205
**Acid-hydrolyzed fat, %**	95.6	95.4	95.3	95.6	0.33	0.8510	0.9930
**Total dietary fiber, %**	60.6^a^	56.7^ab^	53.4^b^	55.6^ab^	1.89	0.0205	0.0475
**Digestible energy^3^, kcal/g**	4.5	4.5	4.5	4.5	0.03	0.9213	0.6414

1 DMB, Dry matter basis.

2 SEM, Standard error of the mean.

3 Measured using bomb calorimetry of food and fecal samples.

4 Linear contrast for Control, 4% SDP and 8% SDP diets only.

a-b Means within a row with different superscripts are different (*P* < 0.05).

### Canine fecal characteristics and metabolites

The fecal characteristics and metabolites from dogs fed different treatment diets are presented in Table 5. No significant interaction was observed between treatment and day for any of the parameters measured (*P* > 0.05); therefore, data are presented by main effect of treatment. Fecal pH ranged from 6.0 to 6.3, fecal score from 3.4 to 3.6, and ammonia concentrations from 0.3 to 0.4, with no significant treatment or day effects (*P* > 0.05).

**Table 5.** Fecal fermentative end-product concentrations of dogs fed diets containing 4% or 8% of spray-dried plasma (SDP).

Item (DMB^1^)	Diets				SEM^2^	*P*-value	Linear contrast^3^
	Control	4% SDP coating	4% SDP	8% SDP			
**Fecal pH**	6.3	6.1	6.0	6.2	0.11	0.1878	0.3101
**Fecal Score**	3.5	3.5	3.4	3.6	0.10	0.5433	0.2982
**Ammonia (mg/g)**	0.3	0.3	0.3	0.4	0.11	0.1585	0.8507
**Short-chain fatty acids (µmol/g)**							
**Acetate**	310.0	326.3	320.3	348.3	16.37	0.2258	0.0472
**Propionate**	126.8^b^	169.9^ab^	160.3^ab^	193.8^a^	10.90	0.0001	0.0001
**Butyrate**	86.8	80.1	89.3	86.5	6.53	0.7516	0.9722
**Total SCFA**	523.6^b^	576.2^ab^	569.9^ab^	628.5^a^	28.34	0.0338	0.0039
**Branched-chain fatty acids (µmol/g)**							
**Isobutyrate**	10.4	11.4	8.2	14.1	1.59	0.0896	0.1385
**Isovalerate**	20.4	19.2	20.1	21.1	1.90	0.8951	0.7810
**Valerate**	12.4	15.1	21.7	20.5	7.09	0.2178	0.0798
**Total BCFA**	43.2	45.7	50.0	55.8	8.42	0.2170	0.0761
**Phenols/indoles (µmol/g)**							
**Phenol**	2.29	1.74	2.34	1.95	0.39	0.3350	0.1969
**4-methylphenol**	0.17	0.18	0.18	0.18	0.03	0.7131	0.5389
**4-ethylphenol**	1.44	1.32	1.38	1.30	0.07	0.2579	0.0986
**Total phenol**	3.90	3.24	3.90	3.43	0.39	0.1640	0.2226
**Indole**	1.36	1.26	1.17	1.27	0.16	0.8295	0.6830
**7-methylindole**	1.04	0.81	0.86	0.73	0.09	0.0650	0.0175
**3-methylindole**	0.42^a^	0.37^ab^	0.37^ab^	0.30^b^	0.04	0.0009	0.0001
**2,3-dimethylindole**	0.04	0.04	0.04	0.05	0.01	0.2273	0.0988
**Total indole**	2.86	2.48	2.43	2.35	0.22	0.1574	0.0343
**Total phenol/indole**	6.75	5.73	6.33	5.78	0.08	0.0839	0.0453

1 DMB, Dry matter basis.

2 SEM, Standard error of the mean.

3 Linear contrast for Control, 4% SDP and 8% SDP diets only.

a-b Means within a row with different superscripts are different (*P* < 0.05).

Among fecal metabolite, only propionate and total SCFA showed significant difference among treatment groups (*P* < 0.05). Dogs fed the 8% SDP diet had higher fecal propionate concentrations (193.8 μmol/g) than dogs fed the Control diet (126.8 μmol/g; *P* < 0.05). Similarly, total SCFA concentrations were higher in 8% SDP group (628.5 μmol/g) compared with Control group (523.6 μmol/g; *P* < 0.05). No treatment effect was observed for acetate (310.0 to 348.3 μmol/g) or butyrate (80.1 to 89.3 μmol/g; *P* > 0.05). However, the linear contrast for acetate was significant (*P* = 0.0472). Overall, total SCFA concentrations were higher in the 8% SDP group, and a significant linear contrast (*P* = 0.0039), suggested that increasing SDP inclusion resulted in greater SCFA production.

For BCFA, concentrations ranged from 43.2 to 55.8 μmol/g, with no treatment or day effects (*P* > 0.05). A trend was observed for valerate and total BCFA, with linear contrasts approaching significance (*P* < 0.1).

Regarding phenol and indole concentrations, only fecal 3-methylindole showed a significant difference among treatment groups (*P* < 0.05). Values were lower in the 8% SDP group (0.30 μmol/g) compared with the Control (0.42 μmol/g), 4% SDP Coating (0.37 μmol/g), and 4% SDP diet (0.37 μmol/g). A strong linear contrast also indicated decreasing concentrations with higher SDP inclusion (*P* < 0.0001). Additionally, fecal 7-methylindole trended towards a treatment effect (*P* = 0.0650), with a significant linear contrast showing a linear decrease as SDP inclusion increased (*P* = 0.0175). Similarly, fecal total indoles showed a linear decrease with increasing SDP (*P* = 0.0343), and total fecal phenol/indole concentrations were also reduced with higher SDP inclusion (*P* = 0.0453).

### Serum chemistry

Serum chemistry was analyzed throughout the study to monitor the health of the dogs and ensure dietary treatments did not cause any negative health effects, and results are shown in Table 6. Significant differences were observed for urea, phosphorus, and triglycerides (*P* < 0.05). Urea concentrations were significantly lower in dogs fed the 4% Coating SDP and 8% SDP diets (17.8 and 16.3 mg/dL, respectively) compared to the Control group (19.8 mg/dL). Phosphorus levels were higher in the 4% Coating SDP group (5.4 mg/dL) compared to the Control (4.7 mg/dL) and 4% SDP group (4.8 mg/dL), and triglyceride concentrations were lowest in the 8% SDP group (54.9 mg/dL) compared to the Control (73.0 mg/dL). Additionally, linear trends were found. Urea showed a significant linear contrast (*P* < 0.0001), with concentrations decreasing as SDP levels increased. Calcium concentrations increased with incremental SDP levels (*P* = 0.0194), with the highest values observed in the 8% SDP group. Alanine aminotransferase also showed a linear decrease (*P* = 0.0233) as SDP inclusion increased. Similarly, triglycerides exhibited a significant linear reduction (*P* = 0.0059), with the lowest values found in the 8% SDP group (54.9 mg/dL) approximately 25% lower than the Control group (73.0 mg/dL). All values remained within the physiological reference range. Total cholesterol approached significance (*P* = 0.0561), showing a trend toward lower cholesterol levels with increasing SDP inclusion, though it did not reach statistical significance.

**Table 6.** Serum chemistry of dogs fed diets containing 4% or 8% of spray-dried porcine plasma (SDP).

Item	**Reference range** ^1^	Control diet	4% SDP Coating diet	4% SDP diet	8% SDP diet	**SEM** ^2^	*P*-value	**Linear contrast** ^4^
**Creatinine, mg/dL**	0.5–1.5	0.6	0.6	0.6	0.6	0.0366	0.1686	0.1368
**BUN^3^ (urea), mg/dL**	6–30	19.8^a^	17.8^b^	18.0^ab^	16.3^b^	1.0916	0.0002	<.0001
**Total protein, g/dL**	5.1–7.0	5.9	6.0	6.0	6.0	0.0916	0.6768	0.7571
**Albumin, g/dL**	2.5–3.8	3.1	3.1	3.1	3.1	0.0716	0.9841	0.8523
**Globulin, g/dL**	2.7–4.4	2.8	2.9	2.8	2.8	0.1071	0.4718	0.6285
**Albumin/globulin ratio**	0.6–1.1	1.2	1.1	1.1	1.1	0.0538	0.1385	0.2336
**Calcium, mg/dL**	7.6–11.4	9.7	10.0	9.8	10.2	0.1454	0.0988	0.0194
**Phosphorus, mg/dL**	2.7–5.2	4.7^b^	5.4^a^	4.8^b^	5.0^ab^	0.1902	0.0149	0.0808
**Sodium, mg/dL**	141–152	146.3	146.7	146.3	146.0	0.4119	0.5084	0.5632
**Potassium, mmol/L**	3.9–5.5	4.4	4.3	4.4	4.3	0.0650	0.3372	0.1719
**Na/K Ratio**	28–36	33.3	34.4	33.7	33.8	0.5140	0.2350	0.2706
**Chloride, mmol/L**	107–118	112.5	112.2	112.2	111.8	0.4187	0.4605	0.3225
**Glucose, mg/dL**	68–126	92.8	90.9	87.9	89.9	2.2447	0.4831	0.3582
**Alkaline Phos Total, U/L**	7–92	62.8	69.0	63.7	63.4	8.7892	0.3210	0.5022
**Canine Alkaline Phosphatase, U/L**	0–40	4.9	4.8	4.7	4.9	1.8763	0.8848	1.0000
**Alanine Aminotransferase, U/L**	8–65	44.0	38.8	40.6	37.8	4.6513	0.0898	0.0233
**Gamma-Glutamyl Transferase, U/L**	0–7	3.8	3.7	4.0	3.8	0.3348	0.3774	1.0000
**Total Bilirubin, mg/dL**	0.1–0.3	0.1	0.1	0.1	0.1	0.0113	0.0952	0.0829
**Creatine Phosphokinase, U/L**	26–310	117.8	112.2	114.7	112.1	6.0985	0.8643	0.5010
**Cholesterol total, mg/dL**	129–297	170.6	180.5	178.4	181.3	8.8633	0.2572	0.0561
**Triglycerides, mg/dL**	32–154	73.0^a^	71.0^ab^	62.1^ab^	54.9^b^	5.3700	0.0263	0.0059
**Bicarbonate (TCO_2_), mg/dL**	16–24	20.3	20.3	20.8	20.3	0.4675	0.7610	1.0000
**Anion Gap, mmol/L**	8–25	18.0	18.5	17.8	18.3	0.4624	0.6762	0.6470

1 Reference ranges were provided by the University of Illinois Veterinary Diagnostics Laboratory.

2 SEM, Standard error of the mean.

3 BUN, Blood urea nitrogen.

4 Linear contrast for Control, 4% SDP and 8% SDP diets only.

a-b Means within a row with different superscripts are different (*P* < 0.05).

### Fecal calprotectin and IgA

**Figure 1.**
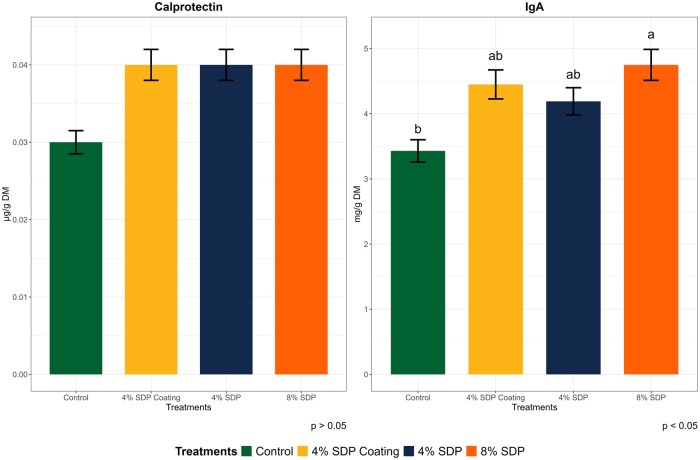
Fecal concentrations of calprotectin and immunoglobulin A (IgA) from dogs fed treatment diets containing 4% or 8% of spray-dried plasma (SDP) in the treatment effect only.

Fecal inflammatory and immune biomarker concentrations in dogs fed different treatment diets are presented in Figure 1. No significant interaction was observed between treatment and day (data not shown; *P* > 0.05). Calprotectin concentrations remained stable across treatments and days, ranging from 0.03 to 0.04 mg/g DM (*P* > 0.05). In contrast, IgA concentrations showed significant differences among treatments (*P* < 0.05), with the highest concentrations in the 8% SDP group (4.8 mg/g DM) compared to the Control group (3.4 mg/g DM). This represents an increase of approximately 39%. Intermediate values were observed for the 4% Coating SDP (4.45 mg/g DM) and 4% SDP (4.19 mg/g DM) groups.

### Fecal microbiota

**Figure 2.**
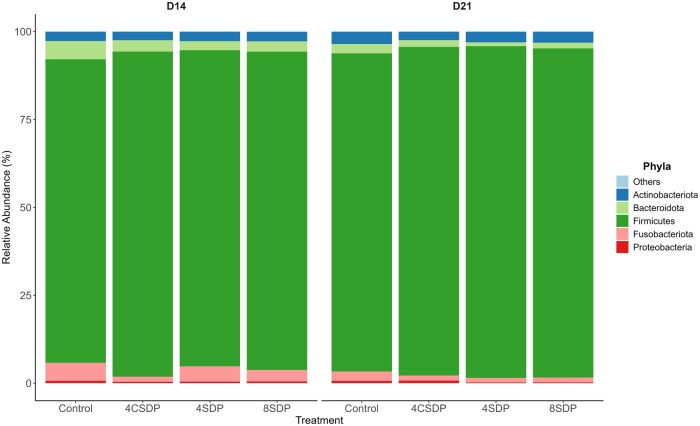
Phyla composition of fecal microbiota from dogs fed treatments diets containing 4% or 8% of spray-dried plasma (SDP). Control: Control diet; 4CSDP: 4% SDP coating diet; 4SDP: 4% SDP diet; 8SDP: 8% SDP diet. *P*-values > 0.5 for all treatments.

**Figure 3.**
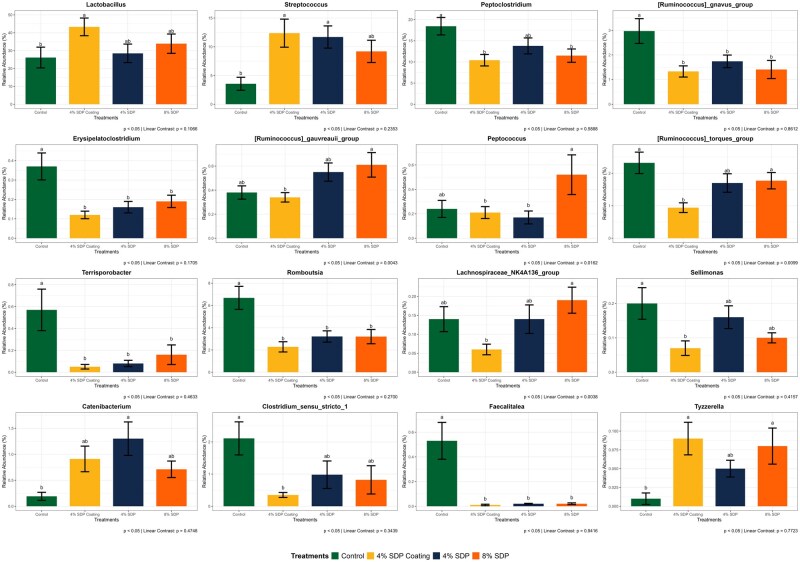
Genera composition of fecal microbiota from dogs fed treatments diets containing 4% or 8% of spray-dried plasma (SDP). Different superscript letters (a, b) within the same genus indicate significant differences among treatments (*P* < 0.05).

A total of 4,425 ASVs (amplicon sequence variants) were identified in the fresh fecal samples. At the phylum level, *Firmicutes, Fusobacteriota, Bacteriodota, Actinobacteriota*, and *Proteobacteria* were the top five most abundant phyla (Figure 2). There was no significant interaction between treatment and day for any phyla (*P* > 0.05). *Firmicutes* were the most dominant across all treatment groups, ranging from 86.9% to 94.2% on days 14 and 21, with a higher relative abundance observed on day 21 across treatments (*P* < 0.05). *Fusobacteriota* showed a significant day effect (*P* < 0.05), decreasing from approximately 5.0% on day 14 to 2.0% on day 21 across treatments. *Bacteriodota* also showed a significant day effect (*P* < 0.05), with relative abundance decreasing from 3.0% to 4.9% on day 14 to 1.2%–2.0% on day 21. *Actinobacteriota* and *Proteobacteria* represented <4% and <1% of the total community, respectively, and did not differ by treatment or day (*P* > 0.05). At the genus level, no significant interaction between treatment and day was observed for any genera (*P* > 0.05). However, several genera were affected by treatment (*P* < 0.05). *Lactobacillus* had the greatest relative abundance in the 4% Coating SDP group compared to the Control. *Streptococcus* was more prevalent in the 4% SDP group compared to the Control. In contrast, *Peptoclostridium, Ruminococcus gnavus group, Erysipelatoclostridium, Terrisporobacter, Romboutsia, Sellimonas, Clostridium sensu stricto 1*, and *Faecalitalea* were more abundant in the Control group relative to the SDP groups. The *Ruminococcus gauvreauii group* and *Lachnospiraceae NK4A136 group* had higher relative abundances in the 8% SDP group compared to the 4% Coating SDP group, and both showed a significant linear increase with increasing SDP levels (*P* = 0.0043 and *P* = 0.0038, respectively). *Peptococcus* showed the highest abundance in the 8% SDP group compared to the 4% SDP group, and its relative abundance also increased linearly with increasing SDP inclusion (*P* = 0.0162). *Ruminococcus torques group* was more prevalent in the Control (2.4%–3.5%) and 8% SDP (1.2%–1.7%) groups than in the 4% Coating SDP group (1.3%–1.4%), and its linear contrast was also significant (*P* = 0.0099). *Catenibacterium* had a greater relative abundance in the 4% SDP group compared to the Control. *Tyzzerella* had higher relative abundance in the 4% Coating SDP and 8% SDP group than the Control group (Figure 3).

**Figure 4.**
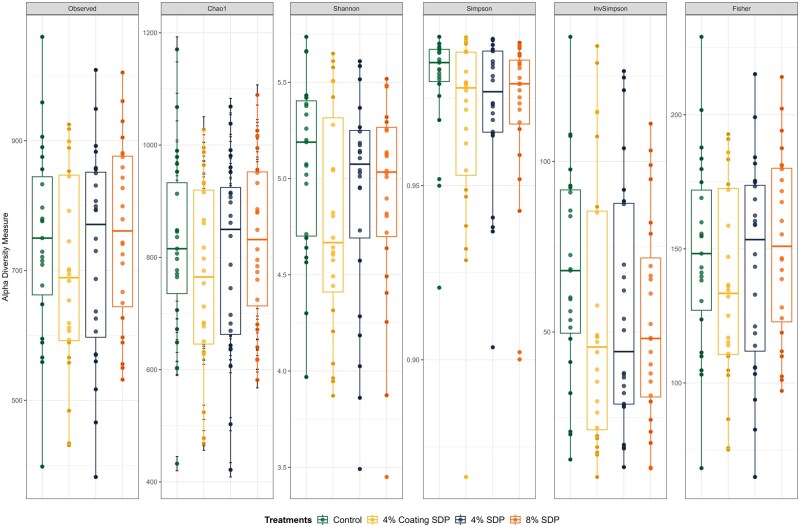
Alpha diversity of fecal microbiota from dogs fed treatments diets containing 4% or 8% of spray-dried plasma (SDP).

**Figure 5.**
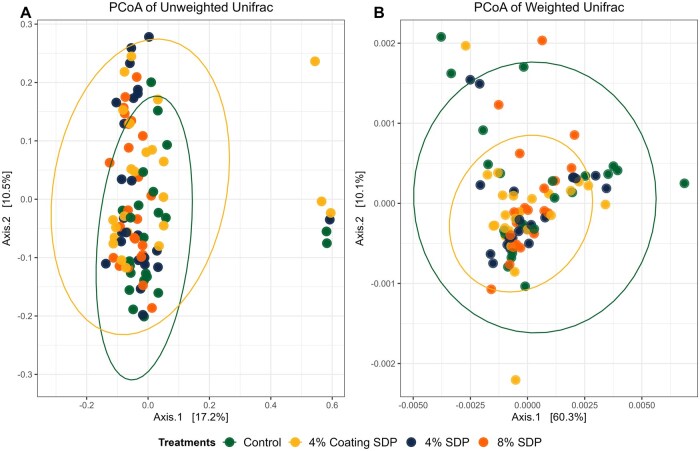
Beta diversity of fecal microbiota from dogs fed treatments diets containing 4% or 8% of spray-dried plasma (SDP) using unweighted (A) and weighted (B) Unifrac matrices.

There was no difference (*P* > 0.05) in alpha diversity among treatment groups across the days according to any of the analyses (Figure 4). Principal coordinates analysis (PCoA) plots using the unweighted (Figure 5A) and weighted Unifrac (Figure 5B) matrices show that the control group cluster separately from the SDP-treated groups (*P*-value = 0.002; *q*-value = 0.0060 for control group vs 4% Coating SDP and 8% SDP group; *q*-value = 0.008 for control group vs 4% SDP), particularly in the unweighted analysis. In the weighted Unifrac PCoA, the clustering is less pronounced, suggesting that although differences exist, the abundance of shared taxa may reduce the separation between the groups.

**Figure 6.**
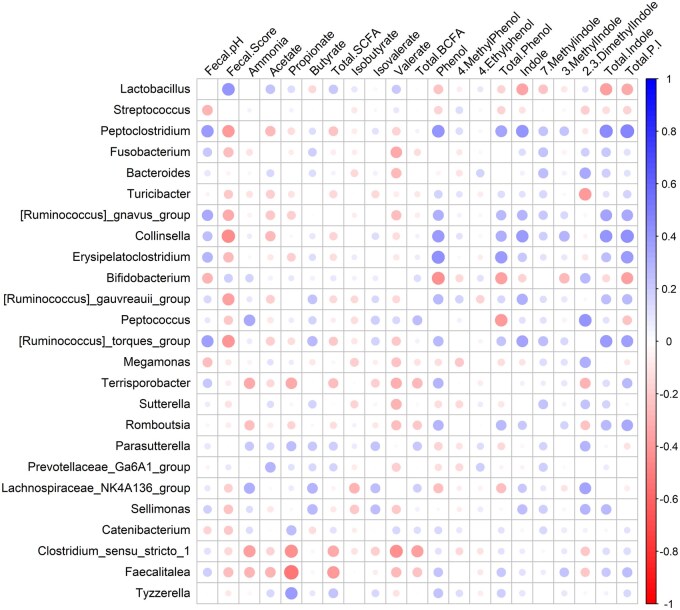
Spearman correlation of fecal metabolites and microbiota taxa in dogs fed treatments diets containing 4% or 8% of spray-dried plasma (SDP). Circle size and color intensity represent the strength and direction of the correlation (blue = positive, red = negative; scale from −1 to 1).

The correlations between fecal metabolites and microbiota taxa in dogs fed the 4% and 8% SDP diets are shown in Figure 6. Fecal propionate and total SCFA concentrations were negatively associated with *Terrisporobacter*, *Clostridium sensu stricto 1*, and *Faecalitalea.* Acetate and total SCFA concentrations showed a slight positive correlation with *Lactobacillus*, while it is negatively linked to several genera, including *Fusobacterium, Bacteroides, Ruminococcus gnavus group, Ruminococcus torques group, Megamonas, Terrisporobacter, Sutterella, Romboutsia, Clostridium sensu stricto 1*, and *Faecalitalea*.

Phenol, total phenol, indole, total indole, and total phenol/indole concentrations are strongly positively correlated with *Peptoclostridium, Ruminococcus gnavus group, Collinsella, Erysipelatoclostridium, Ruminococcus torques group*, and *Romboutsia*, while all show negative correlations with *Lactobacillus* and *Bifidobacterium*. Additionally, total phenol and total phenol/indole concentrations are negatively correlated with *Peptococcus*. 2,3-Dimethylindole is negatively correlated with *Turicibacter* and *Terrisporobacter*, but strongly positively correlated with *Bacteroides*, *Peptococcus*, *Megamonas*, *Sutterella*, *Parasutterella*, *Sellimonas*, and *Lachnospiraceae NK4A136*.

## Discussion

Spray-dried plasma is a by-product that contains high concentrations of essential amino acids and bioactive compounds, such as immunoglobulins, bioactive peptides, metalloproteins and enzymes. Due to these characteristics, this ingredient may show immunomodulatory, anti-inflammatory, and prebiotic-like properties (Campbell et al., 2019; Garcia-Just et al., 2020; Moretó et al., 2020). Recently, the amino acid profile and standardized amino acid digestibility of this ingredient has been evaluated by Khadour et al. (2022). However, in dry dog foods, the dose and method of SDP inclusion—either before extrusion or as a coating—can influence its effectiveness due to the high temperatures and pressure required during the extrusion process.

Due to its potential to support intestinal health in animals, some authors have observed that pre-extrusion inclusion and surface coating of SDP in dog diets improved the ATTD of DM, OM, CP, and TDF (Quigley et al., 2004; de Andrade et al., 2019). However, in this study, a reduction in the ATTD fiber was observed for the 4% SDP diet compared to the Control. Quigley et al. (2004), when evaluating increasing levels of SDP (0% to 3%) in dog diets, observed a quadratic effect on total dietary fiber digestibility, with the peak at 1% inclusion followed by a decline at higher levels. Diverging results might reflect differences in diet formulation, ingredient and nutrient composition, and processing methods.

Protein ingredients typically do not influence alpha diversity; however, they can lead to shifts in beta diversity (Lang et al., 2018). In this study, differences in beta diversity were observed between the Control treatment and the treatments containing SDP, demonstrating a greater similarity in the microbiome among the treatments with plasma inclusion (Martínez-López et al., 2021; Pinto et al., 2022). This effect may be related to bioactive compounds in SDP that modulate microbial metabolism and contribute to a more balanced community structure.

In different species, SDP has been observed to exhibit prebiotic-like effects. However, unlike prebiotics, the purpose of SDP inclusion is not for it to be fermented by microorganisms but rather to contribute to improved intestinal health due to its high concentration of amino acids, immunoglobulins, enzymes, and bioactive peptides present in its composition (Tran et al., 2018; Tapia-Paniagua et al., 2020; Blue et al., 2023). Nutritionally, SDP is particularly rich in lysine, leucine, threonine, valine, and phenylalanine + tyrosine, although isoleucine and the sulfur amino acids (methionine + cystine) are limiting when compared with whole egg powder (Kazimierska and Biel, 2023). In rats, the administration of 8% SDP for 14 d was associated with a reduction in the genus *Bifidobacterium* and an increase in *Blautia*, *Lactobacillus*, *Pedobacter*, *Johnsonella*, and *Pediococcus* (Moretó et al., 2020). In this study, the prevalence of the genus *Lactobacillus* was observed in the treatment with 4% SDP Coating diet. Some species within this genus are responsible for lactic acid production, which is in turn associated with the production of SCFA, such as acetate and propionate (Pot et al., 2014), which in this study were positively correlated with this genus.

The method of inclusion of this ingredient may affect its activity in the organisms due to the thermal sensitivity of its active compounds, which may explain the differences observed between the treatments with SDP inclusion before extrusion and as a coating. During extrusion, protein ingredients can interact with carbohydrates, leading to the formation of Maillard reaction products, which affects their structure and consequently their biological activity. However, there is a void in studies that have evaluated the effects of extrusion on SDP (Vasconcellos et al., 2023).

The majority of the abundant genera in the Control group, such as *Peptoclostridium*, *Ruminococcus gnavus group*, *Ruminococcus torques group*, and *Erysipelatoclostridium*, were positively correlated with the production of phenol and indole, which are putrefactive compounds produced through protein fermentation (Nery et al., 2012; Bastos et al., 2023). The Control group also showed a higher concentration of 3-methylindole compared with the 8% SDP diet. Additionally, a linear decreasing effect was observed for 7-methylindole, 3-methylindole, total indoles, and total phenols/indoles when comparing the Control group (0%) to the other treatments with SDP inclusion before extrusion. This reinforces the SDP can beneficially shift the gut microbiome in healthy adult dogs, as plasma-fed groups showed reduced relative abundance of genera associated with protein fermentation and production of putrefactive compounds (e.g., indoles and phenols).

The inclusion of 8% SDP diet increased the abundance of bacterial genera related to fecal ammonia production, such as *Lachnospiraceae NK4A136 group* and *Peptococcus*. These genera have been associated with protein fermentation and ammonia production, which, when excessive, may have adverse effects on the animal’s health (Clark et al., 2023; Sieja et al., 2023). However, they are also known SCFA producers, which may confer intestinal benefits. In the present study, fecal ammonia concentrations remained unchanged, suggesting no adverse effects related to nitrogenous metabolites. Conversely, dogs on 8% SDP diet exhibited a higher abundance of the genus *Tyzzerella*, which in this study showed a strong correlation with the production of SCFA, particularly propionate. Moreover, the 8% inclusion of SDP also resulted in higher fecal concentrations of total SCFA and propionate compared with Control. Similarly, the genus *Catenibacterium* is also associated with the production of SCFA, particularly propionate (Hiney et al., 2024). In this study, a higher abundance of this taxon was observed with the inclusion of 4% SDP diet, followed by the inclusion of 4% SDP Coating diet, and then the inclusion of 8% SDP diet. These findings indicate that while most parameters were not affected by the method of SDP inclusion (coating vs. extrusion), specific microbial shifts were observed, such as increased abundance of *Lactobacillus* and *Streptococcus* (considered beneficial taxa) and reduced abundance of genera linked to proteolytic fermentation (e.g., *Ruminococcus gnavus group*), suggesting that both method and level of inclusion can influence microbial outcomes.

Interestingly, some microbial responses were more evident in dogs fed the 4% coated diet compared with the same inclusion level applied pre-extrusion. While the underlying mechanism cannot be confirmed, one possible explanation is that post-extrusion application may better preserve certain bioactive properties of SDP that influence microbial metabolism. As the stability of immunoglobulins and other bioactive components was not assessed, future studies should include direct analyses of diet composition to clarify these effects.

Spray-dried plasma may also play an anti-inflammatory role through the production of cytokines and chemokines that regulate inflammation, such as IL-10, IL-4, and TGF-β (Balan et al., 2011; Miró et al., 2023). In this study, consumption of the 8% SDP diet resulted in an increase in fecal IgA concentration compared with the Control group. A similar effect has been observed in poultry and pigs (Gómez-Verduzco et al., 2023). While this effect may be partly attributed to the bioactive components of SDP, we cannot exclude potential contributions from other dietary factors such as fiber and protein composition. IgA production plays an important role in maintaining intestinal homeostasis and inflammatory response against pathogens by inhibiting the adhesion, colonization, and penetration of pathogenic bacteria (Kraehenbuhl and Neutra, 1992; Hand and Reboldi, 2021; Lee et al., 2022).

Spray-dried plasma can also act as a source of essential macro- and microminerals for dogs, providing macrominerals such as phosphorus, calcium, sodium, and potassium, as well as microminerals including iron, zinc, and copper (Kazimierska and Biel, 2023). Among these, phosphorus and calcium stand out due to their higher digestibility and the positive relationship between absorption and increasing doses of SDP, compared with other protein sources (Rodríguez et al., 2016; Munoz et al., 2020). In the present study, although phosphorus digestibility was not directly assessed, serum phosphorus concentrations were higher in dogs fed the 4% SDP Coating diet than in those fed the Control or 4% SDP diet. This difference may reflect reduced phosphorus bioavailability in the 4% SDP diet due to potential complexation during extrusion with other dietary components such as starch or proteins. Interestingly, a higher inclusion level of SDP (8%) appeared to restore serum phosphorus concentrations to levels similar to those observed in the 4% Coating group. While some studies have associated extrusion with increased mineral bioavailability, most of these focused on inorganic rather than organic minerals, such as those present in SDP (Amezcua and Parsons, 2007; Singh et al., 2007; Kraler et al., 2014; Gulati et al., 2020). Importantly, all serum phosphorus values remained within the physiological reference range, and the observed differences were of small magnitude, suggesting no adverse clinical implications. Nonetheless, as serum phosphorus is tightly regulated, these findings warrant cautious interpretation and further investigation to clarify the effects of processing on phosphorus utilization from SDP. For calcium, a linear increase was observed across Control and SDP diets prior to extrusion, although all values remained within physiological reference range.

Some protein ingredients, such as SDP, can influence the production of nitrogenous compounds in the intestine, such as ammonia, and in the blood, such as blood urea nitrogen (BUN) (Hu et al., 2014; Zhang et al., 2023; Bailey et al., 2024). The latter is a marker commonly used to evaluate renal function, specifically the kidneys’ ability to excrete urea (nitrogen) through urine formation (Weiner et al., 2015). Additionally, BUN can indicate how efficiently the animal utilizes amino acids, with lower values suggesting improved utilization (Kohn et al., 2005; Hu et al., 2014; Bailey et al., 2024). In this study, all treatments showed BUN values within the reference range for healthy dogs. A linear decrease in BUN was observed with increasing levels of SDP. A reduction in BUN was also noted for the 4% SDP Coating diet and 8% SDP diet compared with the other two treatments. Although amino acid digestibility was not directly measured in this study, the reduction in BUN may indicate improved nitrogen utilization in dogs fed SDP-containing diets. These observations warrant further investigation to clarify the mechanisms involved. Similar reductions in BUN and improvements in amino acid digestibility have been reported in poultry and swine (Hu et al., 2014; Jeong et al., 2016; Khadour et al., 2022; Bailey et al., 2024).

The serum triglyceride concentration was also affected by the inclusion of SDP. A linear effect was observed among the Control and SDP inclusion, with a significant difference between the mean values of the Control and the 8% SDP groups. Despite these differences, all values remained within the reference range. Hu et al. (2014) also observed a similar effect in pigs supplemented with 4% SDP. In dogs, the combination of prebiotics and SDP has also been shown to alter serum triglyceride concentrations. Although that effect was primarily attributed to fermentable fibers, the presence of SDP may have contributed as well (Lee et al., 2022). The precise mechanisms by which SDP influences lipid metabolism remain unclear; however, evidence from swine studies suggests that SDP may modulate insulin secretion or sensitivity, potentially impacting lipid synthesis and clearance. Insulin regulates lipid metabolism by stimulating lipogenesis and suppressing lipolysis. Therefore, alterations in insulin signaling induced by SDP could partially explain the observed changes in serum triglycerides (de Rodas et al., 1995; Gao et al., 2011). These findings warrant further investigation to clarify the underlying mechanisms.

Overall, the inclusion of SDP in extruded canine diets supported favorable changes in nutrient utilization, microbial metabolism, and immune function, highlighting its potential as a functional ingredient for pet foods. However, some limitations should be acknowledged to aid in the interpretation of these findings. The limited sample size and short feeding duration (21 d/treatment) restrict the ability to evaluate long-term responses or subtle treatment effects. In addition, immune responses were assessed using only fecal IgA and calprotectin, which provide valuable but limited insight into overall mucosal and systemic immunity. Another limitation is that amino acid digestibility was not directly determined at the ileal level in dogs, as such procedures are not feasible due to ethical constraints. However, the use of precision-fed cecectomized roosters has been validated as a suitable model, showing a high correlation with canine amino acid digestibility values (Johnson et al., 1998) and could complement future investigations. Despite these limitations, the present findings provide important evidence of the functional properties of SDP in canine nutrition.

## Conclusion

The incorporation of SDP into canine diets influenced gut health, inflammatory and immune biomarkers, and metabolic regulation while maintaining overall nutrient digestibility. The 8% SDP diet increased fecal SCFA concentrations, particularly propionate, and reduced protein fermentation byproducts, suggesting a beneficial microbial shift. Additionally, SDP modulated the fecal microbiota, increasing beneficial bacterial populations and altering beta diversity. Higher fecal IgA levels in the 8% SDP group indicate immunomodulatory effects, while serum chemistry results suggest improved nitrogen utilization and potential metabolic benefits. The potential effects of extrusion on the functional properties of SDP highlight the importance of evaluating processing methods to maximize its benefits. These findings support the potential of SDP as a functional ingredient in canine diets, providing nutritional and health benefits without compromising digestibility, providing insights for pet food formulations.

## Abbreviations:

AAFCO: Association of American Feed Control Officials
AHF: acid-hydrolyzed fat
ASV: amplicon sequence variant
ATTD: apparent total tract digestibility
BCFA: branched-chain fatty acid
BUN: blood urea nitrogen
CP: crude protein
DMB: dry matter basis
DM: dry matter
EDTA: ethylenediaminetetraacetic acid
GE: gross energy
IgA: immunoglobulin A
NRC: National Research Council
OM: organic matter
PCoA: principal coordinate analysis
SCFA: short-chain fatty acid
SDP: spray-dried plasma
SEM: standard error of the mean
TDF: total dietary fiber
